# Nutritional Care in a Nursing Home in Italy

**DOI:** 10.1371/journal.pone.0055804

**Published:** 2013-02-06

**Authors:** Lorenzo Maria Donini, Barbara Neri, Stefania De Chiara, Eleonora Poggiogalle, Maurizio Muscaritoli

**Affiliations:** 1 Experimental Medicine Department, Sapienza University of Rome, Rome, Italy; 2 Department of Clinical Medicine, Sapienza University of Rome, Rome, Italy; 3 Rehabilitation Clinical Institute “Villa delle Querce” – Nemi, Rome, Italy; Federal University of Rio de Janeiro, Brazil

## Abstract

**Introduction:**

Malnutrition is a clinical condition due to the imbalance among needs, intake and use of nutrients, leading to the increase of morbidity and mortality, and to the impairment of quality of life. Even in industrialized countries undernutrition is becoming an alarming phenomenon, especially involving elderly institutionalized subjects.

A multicentric study called PIMAI (Project Iatrogenic MAlnutrition in Italy), was carried out in Italy over 2005. The aims of this study were to determine the prevalence of malnutrition in hospitals and in nursing care homes (NH), to assess the level of nutritional attention and to measure the perceived quality in food and nutritional care.

This paper represents a preliminary analysis of data collected in a NH included in the PIMAI project.

**Materials and methods:**

A total of 100 subjects (29 males and 71 females, aged 80.2±10 years), were recruited from January to June 2005 at the Clinical Rehabilitation Institute “Villa delle Querce” in Nemi (Rome), among patients in the NH facility. All the participants underwent a multidimensional geriatric evaluation (considering nutritional, clinical, functional and cognitive parameters), and a survey on “perceived quality” of nutritional care.

**Results and discussion:**

According to nutritional status defined by the Mini Nutritional Assessment®, data analysis showed a high prevalence of malnutrition (36%) especially related to advanced age, chewing, cognitive and functional impairments. Patients seemed to consider nutrition to be important for their health; on the other hand, they were not thoroughly satisfied with the quality of food. Particularly, it was observed scarce attention to nutritional status from medical and nursing staff.

**Conclusions:**

Our study confirms the need to pay greater attention to nutritional status in elderly institutionalized subjects. Medical and nursing teams need to be aware of the importance to perform an evaluation of nutritional status in these subset of subjects.

## Introduction

Protein- energy malnutrition (PEM) is a clinical condition due to the imbalance between needs, intake and utililization of nutrients, leading to increased morbidity and mortality, and to the impairment of quality of life [1]. PEM is an alarming phenomenon that involves especially some categories such as elderly people, cancer patients, surgical patients and patients with acute and chronic organ failure and neurological diseases [2]. Prevalence of PEM is high even in industrialized countries, where it affects overall elderly people, increasing from 4% in the early nineties to 15% nowadays. It has been shown that over eighty -year old, hospitalized patients have a chance to develop malnutrition that is 5 times higher than patients who are younger than fifty years; moreover, elderly patients show a lower response to the treatment of PEM [3].

In Europe, considering the time of admission to hospital, prevalence of PEM ranges from 10 to 80%, with an average value of 35% [4] upon hospital admission, and it tends to worsen in most cases during the hospitalization [5], while in long- term care settings and in nursing homes the average prevalence is 30% [6]–[8].

PEM is not easy to manage in elderly patients because of their frailty due to comorbidities, disability and socio- economic problems [9]–[10].

In spite of the recommendations delivered by Scientific Societies and the European Community [11], data from the literature show that in 62–70% of cases malnutrition is not recognized on admission to hospital [12], [13], although a number of tools is available and validated for the geriatric age [14], [15].

Moreover, in elderly patients the effectiveness of nutritional interventions is reduced and the recovery from malnutrition is difficult to achieve [16]–[19]. The incidence of complications in malnourished people in nursing homes is 27% compared to 16% in well- nourished patients, while mortality is three times higher (12.4 vs. 4.7%) [20]. In fact, malnutrition affects the function of organs and systems [2], it decreases muscle mass and muscle strength promoting disability [21]; it impairs the immune response [22] increasing the incidence of hospital infections [23], increases the incidence of thrombosis [24], and delays healing of surgical wounds and pressure sores [25]. Depression and cognitive status impairment are also negatively affected by PEM, in turn triggering a vicious circle in which the subsequent loss of interest for food ends up in worsening the nutritional status [6], [26]–[29]. The deadly combination of above-mentioned consequences of PEM in turn determine a greater need for care, a longer hospital stay, a delay in the recovery from diseases and disability [30] and an increased rate of hospital readmissions [31].

Hence, malnutrition implies both direct and indirect high costs for National Health Services, the former linked to the condition itself, the latter due to the increased vulnerability, to repeated hospitalizations and to social reasons [32]–[33].

On the basis of all these remarks, a multicentric study called PIMAI (Project for Iatrogenic MAlnutrition in Italy), was carried out in Italy in 2005. The aims of the PIMAI study were to determine the prevalence of malnutrition in hospitals and in nursing care homes (NH), to assess the level of nutritional attention and to measure the perceived quality of food and nutritional care.

Data related to the hospitals involved in the PIMAI study were analysed and published [34], while this paper represents a preliminary analysis related to the data collected in a NH included in the PIMAI project.

## Materials and Methods

The study was performed in 100 subjects (29 men and 71 women, aged 80.2±10 years), who were recruited from January to June 2005 among resident patients in the nursing home facility at the Clinical Rehabilitation Institute “Villa delle Querce” in Nemi (Rome), which was one of the eleven PIMAI selected nursing homes throughout Italy. This facility is a level III nursing home, where, according to Italian Health Service organization, only patients with more severe functional impairment can be admitted.

The study was approved by the Ethical Committee of the PIMAI coordinating centre (Regional General Hospital of Bolzano, Italy) and written informed consent was obtained by participants or their legally authorized representatives.

Random sampling from the daily list of new admissions was managed. All the subjects were considered eligible if they agreed to participate in the survey. Patients who presented with oedema, severe hepatic or renal failure, sepsis, or hypothyroidism, were excluded.

The characteristics of the subjects enrolled are reported in Table 1.

**Table 1:** Demographic, clinical, nutritional and functional characteristics of the study participants.

			M	F	p
Subjects		N	29	71	
Age		years	77±10	82.7±9	0.007
Demographic data	Civil status (%)	Single	39,3	31,8	0,001
		Married	21,4	3	
		Divorced	7,1	0	
		Widower	32,1	65,2	
	Education level (%)	Illiterate	4.3	0	0.05
		Elementary	8.7	40	
		Media lower	56.5	44	
		Media higher	13	10	
		Degree	17.3	6	
Clinical status	Comorbidity class (%)	I	44.8	25.7	NS
		II	44.2	62.9	
		III	10.3	11.4	
	Drugs (n)		3.6±3	3.2±2	NS
	Karnofsky (score)		61±19	49,6±15	0.02
Nutritional status	MNA (%)	Normal	17,2	18,3	NS
		Risk of malnutrition	51,7	43,7	
		Malnutrition	31	38	
	Weight loss in the last 3 months (%)	none	35.7	29.6	NS
		1–3 kg	7.1	9.9	
		>3 kg	3.6	4.2	
		Do not know	53.6	56.3	
	Weight loss in the last month (%)	none	40.7	31.4	NS
		1–3 kg	0	7.1	
		>3 kg	3.7	1.4	
		Do not know	55.6	60	
	BMI (%)	<18.5 kg/m^2^	0	6.7	NS
		18-5-24.9 kg/m^2^	50	30	
		25–29.9 kg/m^2^	25	26.7	
		≥30 kg/m^2^	25	36.6	
Cognitive status	SPMSQ (%)	No cognitive impairment (<3 errors)	34.5	21.7	NS
		Mild impairment (3–4 errors)	10,3	14,5	
		Moderate impairment (5–7 errors)	17,2	23,2	
		Severe impairment (>7 errors)	37,9	40,6	
Depression	GDS (%)	>5	47,4	76,7	0.02
Disability	IADL (%)	No impairment (score 11–15)	20.7	1,4	0,001
		Mild impairment (score 6–10)	20,7	14.1	
		Severe impairment (score 0–5)	58,6	84,5	
	ADL	Lost functions/6	2.5±2	3.5±2	NS

Legend: MNA**®**: Mini Nutritional Assessment; BMI: Body Mass Index; GDS: Geriatric Depression Scale; SPMSQ: Short Portable Mental Status Questionnaire; IADL Instrumental Activities of Daily Living; ADL: Activities of Daily Living.

All the enrolled subjects underwent a multidimensional evaluation, including the following aspects and parameters:

1. demographic, social and cultural parameters: gender, age, civil status and education level;
2. the nutritional status:
  - Mini Nutritional Assessment (MNA®) was administered [15]. Nutritional status was defined using the MNA threshold values:
    - normal nutritional status: MNA score ≥24;
    - risk of malnutrition: 17≤MNA score <23.5;
    - malnutrition: MNA score <17;
  - anthropometric parameters: body weight, height, arm circumference (AC), triceps skinfold thickness (TSF), calf circumference (CC), knee height (KH).^.^ In bedridden patients, body weight was measured using a bed- scale, while the height was estimated through KH.
    The body mass index (BMI = weight in Kg/height in m^2^), muscle arm circumference [MAC = (AC- TSF)×π], and stature for bedridden subjects [stature = 94.87−(1.58×KH)−(0.23×age)+4.8 for men] were calculated [35].
    Anthropometric measurements were performed following the procedures described in the “Anthropometric standardization reference manual” [36]. Anthropometric data collection was preceded by an inter- assessor alignment training session. The same tools were used in all the facilities involved in the PIMAI project: a SECA scale 86 (200 kg, to an accuracy of 100 g, certified and homologated as class III), a flexible metallic tape (200 cm, to an accuracy of 1 cm), a telescopic stadiometer (200 cm; 49 cm of telescopic arm), a Holtain Tanner Whitehouse plicometer, and an anthropometer with a graduated scale in centimeters.
  - Muscle strength of the flexor muscles of the forearm, expressed in kg was measured using the Jamar hydraulic dynamometer performing the handgrip strength test on the dominant arm and following standardized procedures [37].
  - A 2-day dietary recall was recorded by a dietician using the weighted average of the major food categories of macronutrients provided by the Italian National Institute for Research on Food and Nutrition (INRAN) [38].
  - The presence of partial or total edentulism and the presence and effectiveness of any dental prosthesis.
  - The degree of impairment of normal food intake according to the following scale:
    - normal orexia (3 or more meals per day): 9–10 points;
    - reduced orexia (if half a served amount was consumed): from 8 to 6 points;
    - poor orexia (if most of the meal was refused) from 5 to 3 points;
    - anorexia (unable to eat anything in 4 consecutive meals): <3 points.
3. Clinical status:
  - The comorbidity level was assessed by the Geriatric Index of Comorbidity [39] consisting in 4 items :
    - Class I: patients with one or more asymptomatic diseases at a subclinical stage or successfully treated diseases in the past;
    - Class II: one or more diseases with mild to moderate symptoms controlled by treatment;
    - Class III: one or more diseases with severe symptoms poorly controlled by treatment;
    - Class IV: one or more of these diseases at the maximum level of severity, not controlled by treatment;
    The definition of the severity of each disease was determined according to the Index of Disease Severity of Greenfield [40];
  - Past medical history and number of current medications;
  - The presence of symptoms potentially interfering with feeding: gastrointestinal symptoms (constipation, diarrhea, heartburn, nausea, vomiting), and pain, that was defined using the following modified version of the Oswestry Low Back Pain Disability Questionnaire [41]:
    - I can tolerate the pain I have without having to use drugs;
    - the pain is bad, but I can manage without having to take drugs;
    - drugs provides me with complete relief from pain;
    - drugs provides me with moderate relief from pain;
    - drugs provides me with little relief from pain;
    - drugs has no effect on my pain.
4. Cognitive status and depression, respectively assessed using the SPMSQ-Short Portable Mental Status Questionnaire (SPMSQ) [42] and the Geriatric Depression Scale (GDS) [43];
5. Functional abilities were measured using the Karnofsky performance status test [44], the Activities of Daily Living (ADL) test [45] and the Instrumental Activities of Daily Living (IADL) test [46];
6. Laboratory parameters: total protein content, albumin, transferrin, prealbumin, red blood cells count, hemoglobin, hematocrit, mean corpuscular volume, white blood cell count, lymphocytes count, C-Reactive Protein (CRP), mucoproteins, cholesterol, and cholinesterase levels were tested. Biochemical assays were performed at the ICR of “Villa delle Querce Nemi” (Rome) using commercial kits supplied by ABX Italy (Rome). A COBAS- MIRA analyser was used. Blood samples were obtained from the antecubital vein after an overnight fast.
7. A survey about the “perceived quality” of nutritional assistance, food and diet was carried out 3 days after the admission, using a structured interview (table 2), including questions about the quality and quantity of food received, opinions on assistance during the meals and on nutritional advices provided by the health staff.

**Table 2:** Survey on perceived food and nutritional support quality.

PERCEIVED FOOD QUALITY		Strongly agree	Agree	Slightly agree	Do not know	Not very agree	Not completely agree	Strongly disagree
1	Is food important for your health?							
2	Do you think that the offered menu is designed for your health?							
3	Do you think that the meal provided to you is sufficient?							
4	Is the received food palatable?							
5	Is the mealtime agreable?							
6	Is the staff ready to change the menu?							
7	Is the staff ready to give assistance at mealtime?							
8	Are the medical staff explanations clear and satisfying?							

PERCEIVED NUTRITIONAL SUPPORT QUALITY		Yes	No	Do not Know
9	Do you live alone?			
10	Do you have trouble to prepare or get the food?			
11	Have you been weighed?			
12	Has anybody given you information about the adequacy of body weight?			
13	Were you asked if the weight has changed in recent months?			
14	Was your stature measured?			
15	Were you asked about your height?			
16	Was nutritional counselling provided by sanitary staff			

### Data analysis and statistics

Nutritional status was correlated with other clinical and functional potential explanatory variables. After verification of the normal distribution of the variables, parametric tests for comparison of means (Student's t-test and ANOVA) and tests for the evaluation of the frequency distribution (Pearson's χ^2^ test) were performed. Statistical significance was set at p value<0.05.

Data were entered a Microsoft Excel database and analysed using the statistical software SPSS for Windows 10.0 (SPSS Inc. 1989–1999).

## Results

Data analysis included all the 100 subjects recruited between January and June 2005 among resident patients in the nursing home facility at the Clinical Rehabilitation Institute “Villa delle Querce” in Nemi (Rome). Males subjects were slightly but not significantly younger than females (77±10 vs 82.7±9 years, p = 0.07).

Overall, accordingly to MNA®, prevalence of malnutrition was 36%; 46% of subjects were at risk of malnutrition, while the nutritional status was found to be normal only in 18% of participants. The prevalence was similar in both genders. About 12% of subjects lost at least 1 kg of body weight in the last three months, while more than 30% of subjects were obese (table 1).

Accordingly to the nutritional status defined with the MNA®^15^, data analysis showed the following results:

1. social and cultural parameters (table 3): malnourished subjects were older (p<0.05) than the well- nourished counterparts, while a trend, but no statistically significant difference, was found for gender, civil status, and educational level;
2. anthropometric and muscle strength variables (table 4): differences (p<0.05) were found for most of anthropometric variables and muscle strength parameters, that were significantly reduced in malnourished subjects;
3. clinical status (table 5): no difference was found for clinical conditions (comorbidity, number of medications) according to nutritional status;
4. symptoms and functions (table 5): hyporexia/anorexia, edentulism and inefficient dental prosthesis were more frequent in malnourished subjects(p<0.05);
5. cognitive status, autonomy and depression (table 6): in malnourished subjects both cognitive (SPMSQ) and functional (Karnofsky, ADL, and IADL tests) impairments were more frequently observed while no difference was found for the GDS (p<0.05);
6. laboratory parameters (table 7): lower levels of serum total proteins, albumin, red blood cells, haemoglobin, haematocrit and cholinesterase were found out in malnourished subjects (p<0.05). An increase of inflammatory parameters (CRP and mucoproteins) was found, even if not statistically significant for CRP;
7. perceived quality of nutritional care (table 8): no differences were observed related to the nutritional status. Globally, patients considered food important for their health but they were not completely satisfied of the quality of food. They also noticed a low attention paid to nutritional status (e. g. body weight and height measurements, information provided about these parameters and other nutritional aspects) from medical and nursing staff.

**Table 3:** Nutritional status (MNA®) and social-cultural characteristics.

			Nutritional Status			p
			Normal	At risk	Malnutrition	
Gender	M	%	27.8	32.6	25	NS
	F		72.2	67.4	75	
Age		years	73.7±10	81.8±9	83.6±9	0.001
Civil Status	Single	%	50	34.1	25	NS
	Married		5.6	4.5	15.6	
	Divorced		5.6	2.3	0	
	Widower		38.8	59.1	59.4	
Education level	Illiterate	%	0	0	5.6	NS
	Primary school		33.3	29.7	27.8	
	Middle school		33.3	56.8	44.3	
	Secondary school		11.2	8.1	16.7	
	Degree		22.2	5.4	5.6	

Legend: MNA**®**: Mini Nutritional Assessment.

**Table 4:** Anthropometric parameters and nutritional status according to MNA®.

			Nutritional status according to MNA®			
			Normal	At risk	Malnutrition	p
Weight		kg	79.4+17	61.8+14	42.9+4	0.000
BMI		kg/m^2^	32.6±6	26.9±5	19.3±1	0.000
CC		cm	35.8±5	29.8±4	25.±3	0.000
TSF	M	mm	16.8±8	10.3±8	8.1±5	NS
	F	mm	27.7±7	19.1±7	11.5±6	0.000
% subjects with reduced TSF (M< 5.2 mm; F<9.7 mm)*		%	0	7	44.4	0.000
AC	M	cm	30±3	24.8±3.7	22.2±4.71	0.006
	F	cm	31.3±6	25.7±4	21.7±4	0.000
MAC	M	cm	24.7±2	21.6±3	19.6±4	0.036
	F	cm	22.6±4	19.7±3	18.1±3	0.000
% subjects with reduced MAC (M<22 cm; F<18.9 cm)*		%	17.6	53.5	69.4	0.002
strength (dynamometer)	M	kg	20±9	13.7±8	12.2±8	NS
	F	kg	8.8±9	3.1±4	0.9±3	0.003

Legend: MNA**®**: Mini Nutritional Assessment; BMI: Body Mass Index; CC: calf circumference; TSF: Triceps Skinfold Thickness; AC: Arm Circumference; MAC: Muscle arm circumference.

* The weighted mean of 10^th^ percentile values for Italian samples enrolled in the SENECA study were used as lower limits of normality for anthropometric parameters (Euronut SENECA Investigators (1991) Nutritional status – anthropometry. Eur J Clin Nutr 45: S3).

**Table 5:** Clinical conditions according to nutritional status (MNA®).

		Nutritional status(prevalence %)			p
		Normal	At risk	Malnutrition	
Comorbidity *	I	27.8	34.8	28.6	NS
	II	66.6	60.9	48.5	
	III	5.6	4.3	20	
	IV	0	0	2.9	
Gastroenteric symptoms	Nausea	0	0	5.7	NS
	Vomit	0	2.2	2.9	NS
	Diarrhea	0	2.3	11.8	NS
	Constipation	0	4.4	0	NS
	heart burn	0	6.7	2.9	NS
Painful symptoms *	I	55.6	58.7	72.1	NS
	II	33.3	19.6	5.6	
	III	11.1	4.3	5.6	
	IV	0	8.7	2.8	
	V	0	8.7	5.6	
	VI	0	0	8.3	
Appetite	Good	83.3	45.6	39	0.000
	Decreased	16.7	39.2	41.6	
	Scarce	0	15.2	19.4	
	Absent	0	0	0	
Edentulism	Absent	22.2	4.5	0	0.004
	Partial	50	63.6	86.1	
	Total	27.8	31.8	13.9	
Dental prosthesis	Absent	37.5	12.5	5.9	0.002
	Partially efficient	43.8	32.5	17.6	
	Inefficient	18.8	55	76.5	

Legend: MNA®: Mini Nutritional Assessment; Comorbidity: Geriatric Index of Comorbidity^39^ and Index of Disease Severity^40^; Painful symptoms: modified Oswestry Low Back Pain Disability Questionnaire^41^.

**Table 6:** Functional status, cognitive impairment and depression according to nutritional status (defined by MNA®).

			Nutritional status			p
			Normal	At risk	Malnutrition	
SPMSQ		Score	2.59±2	5.24±4	7.83±3	0.000
	No cognitive impairment (<3 errors)	Prevalence (%)	58.8	26.7	8.3	0.000
	Mild impairment (3–4 errors)		17.6	15.6	8.3	
	Moderate impairment (5–7 errors)		23.5	24.4	16.7	
	Severe impairment (>7 errors)		0	33.3	66.7	
GDS	Score		6.29±3	6.79±3	8.12±3	NS
	Prevalence of depression (score >5)	(%)	64.7	64.3	76.5	NS
Karnofsky performance status (score)			70±14	54.78±16	41.94±11	0.000
ADL	Score		10.78±2	6.65±4	1.86±3	0.000
	Lost functions/6		0.39±1	2.65±2	5.22±1	0.000
	No lost functions/6	Prevalence (%)	77.7	26.1	2.8	0.000
	1–2 lost functions/6		16.7	19.6	5.6	
	> 2 lost functions/6		5.6	54.3	91.6	
IADL	Score		7.11±4	3.09±4	0.67±2	0.000
	No impairment (score 11–15)	Prevalence (%)	16.7	8.7	0	0.000
	Mild impairment (score 6–10)		44.4	13	5.6	
	Severe impairment (score 0–5)		38.9	78.3	94.4	

Legend: MNA**®**: Mini Nutritional Assessment; SMPQ: Short Portable Mental Status Questionnaire; GDS: Geriatric Depression Scale; ADL: Activities of Daily Living; IADL: Instrumental Activities of Daily Living.

**Table 7:** Laboratory parameters according to nutritional status (defined by MNA®).

			Nutritional status			
			Normal	At risk	Malnutrition	p
Total Protein		g/dl	6.8±0.7	7.1±0.7	6.4±0.7	0.004
Albumin		g/dl	3.83±0.4	3.66±0.4	3.43±0.5	0.005
	≥3.0 g/dl	% of patients	72.2	60	52.7	NS
	2.9–2.5 g/dl		27.8	33.3	30.6	
	<2.5 g/dl		0	6.7	16.7	
Transferrin		mg/dl	259.4±49	237.5±44	213.5±55	NS
	≥150 mg/dl	% of patients	87.5	84.2	54.2	NS
	149-100		12.5	13.2	41.6	
	<100		0	2.6	4.2	
Prealbumin		mg/dl	25.1±6	21.1±7	21.8±8	NS
Haemachrome	RBC	#/ml	4627.2	4575.8	4263.6	0.03
	Hb	g/dl	14.1±2	13.3±2	12.6±2	0.006
	Ht	%	40.7±4.4	38.6±4.8	36.4±4.5	0.005
	MCV	φ/L	88±3.8	85.7±7.5	85.5±5.4	NS
	WBC	#/ml	6964.7±2191	6793.3±1963	7585±3588	NS
Lymphocytes		#/ml	2300.4±899	2313.8±989	2270.9±725	NS
	>1500 #/ml	% of patients	83.3	86.7	86.1	NS
	1500-1200		16.7	8.9	11.1	
	<1200		0	4.4	2.8	
CRP		mg/dl	8.5±10	10.2±18	15±21	NS
	<7 mg/dl	% of patients	64.7	64.5	47.2	NS
	7–20		23.5	24.4	30.6	
	>20		11.8	11.1	22.2	
Mucoproteins		mg/dl	100.5±31	111.4±38	131.5±51	0.02
	<160 mg/dl	% of patients	94.1	88.9	77.8	NS
	≥160		5.9	11.1	22.2	
Cholesterol		mg/dl	202.8±45	187.1±43	180.4±43	NS
Cholinesterase		U/l	8647.4±2113	7138.2±2067	6190.8±1894	0.00

Legend: MNA**®**: Mini Nutritional Assessment; RBC: red blood cells; Hb: hemoglobin; Ht: hematocrit; MCV: mean cell volume; WBC: white blood cells; CRP C-reactive protein.

**Table 8:** Perceived quality of nutritional care related to nutritional status (MNA®).

				Entire sample	Nutritional status			
					Normal	At risk	Malnutrition	p
1	Is food important for your health ?		Score	1.67±1	1.78±1	1.36±0.5	2.31±0,2	0.02
2	Do you think that the offered menu is designed for your health ?		Score	2.88±2	3.33±2	2.91±2	2.31±1	NS
3	Do you think that the meal provided to you is sufficient ?		Score	2.19±1	2.44±2	2.09±1	2.13±1	NS
4	Is the received food palatable ?		Score	3.32±2	3.39±2	3.60±2	2.63±1	NS
5	Is the mealtime agreable ?		Score	1.99±1	2.33±2	1.83±1	1.94±1	NS
6	Is the staff ready to change the menu ?		Score	2.82±2	2.65±2	2.67±1	3.31±2	NS
7	Is the staff ready to give assistance at mealtime ?		Score	2.21±1	2.76±2	1.68±0.5	2.75±2	0.01
8	Are the medical staff explanations clear and satisfying ?		Score	2.87±2	3.17±2	2.65±2	3.00±2	NS
9	Do you live alone?	Yes	%	21.7	11.1	25.7	25	NS
		No		76.8	83.3	74.3	75	
		Do not know		1.4	5.6	0	0	
10	Do you have trouble to prepare or get the food ?	Yes	%	34.8	38.9	30.6	31.3	NS
		No		63.8	66.7	68.6	50	
		Do not know		1.4	5.6	0	0	
11	Have you been weighed ?	Yes	%	32.9	30.4	30.6	31.3	NS
		No		60	50	61.1	68.8	
		Do not know		7.1	11.1	8.3	0	
12	Has anybody given you information about the adequacy of your weight ?	Yes	%	35.4	20.0	33.3	12.5	NS
		No		64.6	80	66.7	86.5	
13	Were you asked if the weight has changed in recent months ?	Yes	%	25.4	26.7	31.3	12.5	NS
		No		74.6	73.3	68.8	87.5	
14	Was your stature measured ?	Yes	%	31,7	46.7	28.1	25	NS
		No		68.3	53.3	71.9	75	
15	Were you asked for stature ?	Yes	%	19.7	14.3	25.8	12.5	NS
		No		80.3	85.7	72.4	87.5	
16	Was nutritional counselling provided by medical staff?	Yes	%	20.3	18.8	25	12.5	NS
		No		79.7	81.3	75	87.5	

Average score obtained for questions 1–8: answers are quoted from 1 to 7 in a Likert rating scale: strongly agree = 1, agree = 2, slightly agree = 3, do not know = 4, not very agree = 5, not completely agree = 6, strongly disagree = 7.

Legend: MNA**®**: Mini Nutritional Assessment.

## Discussion

Carried out in a nursing home in Italy, as a part of the PIMAI multicentric project, this study showed a high prevalence of malnutrition, related in particular to functional and cognitive impairment. Patients seemed to be aware of the role of nutritional status in their life and they were asking for a more precise nutritional care and an improved quality of catering services.

The ageing process worsens the nutritional status and, consistently with different studies, in our sample malnourished subjects were older than non- malnourished counterparts (83.6±9 vs 73.7±10 years). Regarding social and cultural parameters, loneliness, poverty and a low educational level [47] are considered to be risk factors for anorexia in the elderly [12]. Ramic et al. [48] pointed out that people living in loneliness tend to reduce the number of daily meals and the intake of proteins, fruits and vegetables. The SOLINUT study [49] supports the hypothesis that social isolation is associated with an inadequate caloric intake to cover the normal daily energy requirements. In our study these associations are not completely verified, although a trend is evident: low educational level subjects and widowed patients are more frequently malnourished.

Data in our study confirm that ageing- induced malnutrition is usually secondary to a decrease in appetite and in food intake [50]. Early satiety in elderly people is due to anatomical and functional modifications in the stomach and to changes in the concentration of some hormones involved in appetite control (cholecystokinin and leptin) [51]. Moreover, the impairment of taste and smell, the reduction of visual and auditory acuity and dental problems contribute to reduce food intake in the elderly subjects [52], limiting the capacity to prepare meals [53] and reducing the chewing capability [54].

Regarding difficulties in chewing, we demonstrated that 41.7% of the malnourished subjects had a reduced food intake, 86.1% were partially edentulous and 76.5% had ineffective dental prosthesis. Some studies [55], [56] proposed and demonstrated the existence of a link among dental efficiency, food intake and malnutrition. Subjects with these problems tend to change their dietary habits eliminating all foods that are difficult and too hard to chew (fruits, vegetables and meat) with an inevitable impairment of the nutritional status.

Disability and cognitive impairment are further potential causes of malnutrition [20], [57]–[61].

The level of autonomy was assessed with three instruments: Karnofsky performance status test, activities daily living (ADL) test and instrumental activities of daily living (IADL) test. Malnourished subjects seemed to have a greater level of disability in activities of daily living and with frequent requests of nursing assistance and medical care (as verified by the Karnofsky test score). These data are indirectly confirmed by the handgrip strength test, that showed deeply reduced values in malnourished subjects (3,93±7 vs 11,94±10 kg). These results are in line with existing literature: in most studies, disability is associated to biochemical and anthropometric parameters related to malnutrition, to the need for assistance and home care and to a longer stay in health care facilities [24], [27], [48], [58]–[60], [62]..

Similarly, the mental state may affect nutritional status and cognitive impairment is more common in malnourished than well-nourished subjects [59]. In our sample, the prevalence of malnutrition in severe mental impairment (>7 errors to the SPMSQ) was 66.7% while in subjects at risk of malnutrition and in well-nourished patients the prevalence decreased to 33,3 and 0%, respectively.

In several studies, depression is considered to be a risk factor for malnutrition and vice versa [6], [26]–[29]. Depression in the elderly may be justified by the loss of an active social role, loss of affects, disability and institutionalization. Inconsistently with the literature, in our study, mood was not correlated with nutritional status. However, the prevalence of depression and the score of the GDS showed a tendency to be greater in malnourished patients.

Previous studies of our group highlighted a relationship between nutritional status and comorbidity [62], [63]. In the present sample this association was not confirmed in a statistically significant fashion. However, a trend towards a poorer health status (comorbidity level III/IV) emerged in our malnourished subjects (23 vs 5.6%). Moreover, the prevalence of gastrointestinal symptoms as well as that of pain were higher in malnourished subjects (23,3 vs 0% and16,7 vs 0%, respectively). Furthermore, the laboratory parameters showed significant differences (with regard to proteins, red blood cells and cholinesterase levels) between malnourished and well-nourished subjects. Consistently with previous studies describing an association between inflammation and malnutrition in the elderly, in our sample of malnourished individuals, inflammatory markers were increased, although the rise of CRP levels was not statistically significant; similarly to clinical and comorbidity data in our sample, no significant differences were reported with respect of the nutritional status; we can hypothesize that a potential explanation may be the setting of recruitment: according to the Italian Health Service organization, in the facility patients with a severe functional impairment are usually admitted, with a relative stable and good clinical condition.

Improvement of the quality of life for residents in healthcare facilities may be achieved through the identification of critical points concerning in particular the quality of the catering service and the perceived quality of medical and nursing care [64], [65]. Therefore, an *ad hoc* questionnaire was created for the PIMAI project consisting of sixteen questions focused on the catering service and medical/nursing care dedicated to nutritional aspects. Our data show no statistically significant differences for these parameters in relation to nutritional status. Anyway, while considering food important for their health status, patients were not totally satisfied of the quality of diet in the NH. They also noticed a low attention paid to nutritional status from medical and nursing staff. Few studies in the literature dealt with the surveys on the perceived quality of diet and on the counseling received about nutrition in nursing homes. Lengyel et al. [66] pointed out that NH patients may have a good perceived quality of life, even if they are dissatisfied with the choices of food and the availability of “snacks”. Crogan et al. [67] as well as Carrier and coll. [68] described the utility of questionnaires for assessing perceived quality in health care facilities, that is directly proportional to the quality of life of patients, since adequate food in quality and quantity was essential not to lose weight and avoid protein- energy malnutrition. Noteworthy, the lack of interaction between caregiver and patient as well as the lack of competence of healthcare staff and the inadequate quality and quantity of food were shown to negatively affect food intake [69].

A limitation of the present study was the small sample size. The whole analysis of data collected in the full database of the PIMAI project will give further and more complete information. Another limitation is the setting of enrollment: our subjects were enrolled in a level III nursing home, where, according to Italian Health Service organization, only patients with more severe functional impairment can be admitted.

Finally, the present study stresses the need to pay greater attention to nutritional status in elderly institutionalized subjects. Medical and nursing staff need to be aware of the importance to perform the evaluation of nutritional status (especially anthropometric parameters and eating patterns) in this subset of subjects and to consider, in particular, cognitive and functional performances as risk factors for malnutrition. Patients seem to be aware of the role of nutritional status in their life and they ask for a more precise nutritional care and an improved quality of catering services. Individual factors play a pivotal role in malnutrition; on the other hand it has to be stressed that in nursing homes several organizational aspects work negatively influencing indirectly the onset or the worsening of malnutrition: for example, the amount of nursing staff available during meals to provide feeding assistance is crucial. Hence, multilevel models of analysis should be prompted in order to better evaluate the relevance of organizational features in terms of prevention and care of malnutrition in nursing home residents.

Therefore, the management of hospitals and nursing homes should take into account the quality of catering services as a way to improve nutritional and health status of elderly institutionalized subjects.
